# Analyzing the genetic profile of autistic children and adolescents with minimal verbal abilities

**DOI:** 10.3389/fgene.2026.1647481

**Published:** 2026-02-04

**Authors:** Silvia Guerrera, Ilaria Venezia, Maria Grazia Logrieco, Laura Casula, Rossella Capolino, Maria Cristina Digilio, Maria Lisa Dentici, Marina Macchiaiolo, Federico Casciani, Fabiana Cortellessa, Lorenzo Sinibaldi, Andrea Bartuli, Silvia Di Tommaso, Gemma D'Elia, Viola Alesi, Cristina Roberti, Antonio Novelli, Giovanni Valeri, Stefano Vicari

**Affiliations:** 1 Child Adolescent Neuropsychiatry Unit, Bambino Gesù Children’s Hospital, IRCCS, Rome, Italy; 2 Department of Health Sciences, Division of Child Neuropsychiatry, ASST Santi Paolo e Carlo, San Paolo Hospital, Milan, Italy; 3 Department of Humanities, University of Foggia, Foggia, Italy; 4 Medical Genetics Unit, Bambino Gesù Children’s Hospital, IRCCS, Rome, Italy; 5 Laboratory of Medical Genetics, Translational Cytogenomics Research Unit, Bambino Gesù Children’s Hospital, IRCCS, Rome, Italy; 6 Life Sciences and Public Health Department, Catholic University, Rome, Italy

**Keywords:** autism spectrum disorder, genotype-phenotype correlations, syndromes, minimally verbal, neurodevelopmental disorder

## Abstract

**Introduction:**

Comprehensive care for autistic youth with severe symptoms and language impairment includes genetic testing to find underlying causes. Identifying a genetic diagnosis helps determine prognosis, guide treatment, assess recurrence risk, and connect families with targeted resources and support networks.

**Methods:**

This cross-sectional study analyzed retrospectively data of a cohort of 60 Autism Spectrum Disorder Minimally Verbal (MV) past age 5 children and adolescents who underwent several genetic investigations and were included in an evaluation protocol including cognitive, adaptive, psychiatric, parental stress and autism characteristics’ evaluations to identify whether there were any specific clinical or genetic characteristics in the group of minimally verbal autistic individuals.

**Results:**

The percentage of genetic disorders detected in the series is 22.6%. Two groups of MV autistic individuals were defined: those without a known genetic cause (n = 46, neuropsychological data available for 32 individuals) and those with an associated genetic condition (n = 14, neuropsychological data available for 8 individuals). Most participants in both groups scored below 70 on Nonverbal Intelligence Quotient (NVIQ) (77.5% in the first group *versus* 77.7% in the latter) and adaptive functioning was impaired in both groups, without significant differences. Autism severity, measured by the ADOS-2, was significantly higher in individuals without causative alteration, particularly in Total Comparison Score. However, no differences were found between groups in restricted and repetitive behaviors. CBCL showed high levels of internalizing and externalizing problems in both groups, with no differences. Similarly, parental stress levels were high in both groups.

**Discussion:**

This is the first study analyzing the genotype-phenotype correlation in MV autistic individuals. In this sample, the prevalence of genetic syndromes was found to be twice as high as in the general autistic population (22.6% *versus* 10%). Regarding the autistic characteristics’ severity which appear to be higher in individuals without genetic causative alteration and the absence of significant differences in cognitive, functional and behavioural characteristics, we hypothesised that, in the MV autistic population without genetic causative alteration, there are specific and unknown characteristics of the MV profile which have a greater impact than the individual genetic condition reported.

## Introduction

According to the Diagnostic and Statistical Manual of Mental Disorders, 5th Edition, Text Revision (DSM-5-TR), Autism Spectrum Disorder (ASD) is defined as a neurodevelopmental condition. It is characterized by deficits in social communication and interaction, as well as repetitive and restricted patterns of behavior and interests (e.g., repetitive body movements such as hand flapping, sensory sensitivities, and circumscribed interests) (American Psychiatric Association, 2022). The male sex is widely recognized as one of the most established etiological factors for ASD, with an estimated prevalence of 3.8 times higher among boys than girls (Maenner et al., 2023), leading to the concept of a “female protective effect,” where females may require a greater etiological burden to exhibit the same level of impact as males. The current global prevalence of ASD is estimated to be approximately 1% (Zeidan et al., 2022) and has significantly increased over the last 20 years (Baio et al., 2018). Research conducted over the past 4 decades has revealed not only the substantial variability of the underlying causal factors of autism but also that autism is one of the most heritable conditions (Rutter, 2011). Recent molecular genetic studies have led to the identification of an increasing number of genes associated with autism and have stimulated a growing body of literature on potential biological insights (Vorstman et al., 2017; Vorstman and Scherer, 2023; Vicari et al., 2019; Quesnel-Vallières et al., 2019; Savatt and Myers, 2021). There exists a category of monogenic disorders, such as Rett syndrome, tuberous sclerosis complex (TSC) and fragile X syndrome, which exhibit highly distinctive physical characteristics and may be associated with autism. These monogenic disorders are infrequent, and none of these genes can account for more than 1%–2% of ASD cases; however, collectively, they are estimated to represent about 5% of ASD cases (Sztainberg and Zoghbi, 2016). Some researchers classify these disorders as “syndromic autism”, distinguishing them from “nonsyndromic” autism based solely on clinical criteria. The term “syndromic” denotes conditions in which ASD occurs alongside additional phenotypes and/or dysmorphic traits. In contrast, “nonsyndromic” generally refers to “classic autism,” as characterized by Kanner, in which no additional symptoms are observed (Vorstman and Scherer, 2023). For the majority of nonsyndromic ASD cases, the underlying cause remains unknown, and the term “idiopathic autism” has been alternatively used, although a genetic component has also been acknowledged in these instances (Sandin et al., 2014). These discoveries have propelled research on genomic loci linked to genetic susceptibility for ASD (Weiss et al., 2009; Weiss et al., 2008) and the pursuit of rare and common variants that contribute to the risk of developing ASD (Anney et al., 2012). In the early 2000s, array Comparative Genomic Hybridization (CGH) technology enabled researchers to identify an enrichment of copy number variations, such as duplications and deletions in regions 15q11-13, 16p11.2, 22q11.2, and 7q11.23, in both syndromic and nonsyndromic autistic patients (Sanders et al., 2011). It is evident from molecular genetics studies that autism is not only clinically heterogeneous but also highly diverse in terms of genetic causation at the molecular level. The rare variants associated with autism, like common variants, are pleiotropic. Consequently, there is likely no definitive distinction between rare monogenic and common multifactorial autism (Vorstman and Scherer, 2023). Nevertheless, ASD cannot be entirely elucidated by genetics: the influence of environmental factors has been extensively investigated (Mandy and Lai, 2016), emphasizing that environmental risks and genetic predispositions are interconnected rather than independent factors, linked through gene-environment correlation (Rutter et al., 2006), which can be either passive or active (Thapar and Rutter, 2021).

Beyond global insights into ASD genetics, a growing body of research has investigated the genetic bases of speech and language impairment in autism. Several candidate genes implicated in language development have also been associated with ASD, most notably *FOXP2* and related transcriptional networks influencing cortico-striatal and cerebellar circuits (Vargha-Khadem and Gadian, 2005; Fisher and Scharff, 2009). Mutations in genes such as *CNTNAP2* and *SHANK3* have been linked to both autism and severe speech/language phenotypes, including minimally verbal presentations (Alarcón et al., 2008; Durand et al., 2007). Importantly, animal model studies have provided converging evidence: mouse knockouts of *Shank3*, *Cntnap2*, and *Foxp2* display atypical ultrasonic vocalizations, supporting their role in communication-related pathways (Ey et al., 2012; Peñagarikano et al., 2011; Castellucci et al., 2016; Abdavinejad et al., 2024). These findings suggest that speech and language impairment in autism may arise from partially distinct genetic mechanisms, some of which overlap with broader neurodevelopmental risk loci. However, despite these advances, systematic studies focusing specifically on the genetic underpinnings of minimally verbal autism remain limited.

Delving further, to our knowledge, there is a gap in the current literature regarding the relationship between individuals with ASD who have minimally verbal abilities and genetic factors.

For example, the KAT6A (Arboleda-Tham) syndrome, a Mendelian disorder caused by pathogenic variants in the lysine acetyltransferase 6A (KAT6A) gene (Ng et al., 2024), is characterized by intellectual disability and autism-related features (particularly restricted interests and repetitive behaviors) with an important speech/language impairment (e.g., minimally verbal).

A recent study (Barone et al., 2023) analyzed the autistic phenotype profile in two siblings with PARK2 microdeletion, which appears to be involved in heterogeneous autistic variability, in some cases associated with significant impairment of the language profile.

It also emerges the need to identify objective outcome measures of expressive language for use in children with ASD and minimally verbal abilities as recently investigated in a sample with Phelan-McDermid Syndrome (Rankine et al., 2017). Indeed, the development of verbal language in autistic children differs from neurotypical development and shows wide variability in expression (Tek et al., 2014) with language profiles in autism being variable (Kissine et al., 2023). Around the age of three, 50%–60% of autistic children are still non- or minimally speaking, but by the age of six or seven, around 60%–80% of autistic children develop expressive and receptive structural language, often at levels close or even superior to typical. During this 3- to 7-year of age interval there is a variety of language trajectories, from steady and relatively modest growth curves to surprisingly steep language gains, but after this point, language scores usually progress in relatively predictable fashion. Some authors speculated that many autistic children display an atypical and clinically recognizable trajectory in the acquisition of structural language (phonology, vocabulary and morphosyntax) arguing that this trajectory, called bayonet-shaped development, includes early language onset, regression, a plateau, and a certain level of language recovery (Mottron and Gagnon, 2023). The authors assumed that this developmental trajectory is associated with a prototypical autism phenotype considered as a discernible, rapidly identifiable, unique pattern during the plateau period (period without major language gains, usually in the 2–5-years period). Eventually about 25%–30% of autistic children do not develop or fail to develop functional spoken language and remain *minimally verbal* (MV) past age 5 (Anderson et al., 2007; Norrelgen et al., 2015), with implications for social and adaptive functioning in adulthood (Tager-Flusberg and Kasari, 2013). The usually refers to children who exhibit a limited vocabulary consisting of a small number of spoken words and fixed phrases (Tager-Flusberg and Kasari, 2013). There are studies that define MV on the basis of expressive vocabulary size (e.g., fewer than 20 or 30 words), others by the inability to produce multi-word utterances, or by the persistence of single-word speech beyond a certain age threshold (Kasari et al., 2006; Thurm et al., 2007). Additional approaches adopt parent-report measures or standardized language assessments to determine functional speech abilities (Pickles et al., 2014; Almirall et al., 2016; La Valle et al., 2024). A widely accepted definition of minimally verbal (MV) autistic children is derived from the Autism Diagnostic Observation Schedule, Second Edition (ADOS-2) (Lord et al., 2012). Within this framework, the term “minimally verbal” designates individuals evaluated with Module 1 of the ADOS-2, which is designed for children over 30 months of age who either produce no speech or use only a very limited repertoire of words in simple combinations (Bal et al., 2016; Fok and Bal, 2019; Lord et al., 2012; Thurm et al., 2015; Williams et al., 2018). It is important to note that, the use of standardized assessments commonly applied in language therapy to evaluate the linguistic abilities of MV autistic children has been critically examined, as such measures often present challenges due to the complexity of task instructions and the requirement for active child participation (Schaeffer et al., 2023).

Language in autistic children remains imperfectly understood, and identifying the factors that may predict, favor or shape linguistic trajectories within the crucial 3-to-7 period remains one of the major scientific challenges in autism research (Kissine et al., 2023), including possible associated genetic profiles. Studies have been conducted in the literature to search for predictive factors on language development; for example, Kissine M. and colleagues in recent research (Kissine et al., 2023) speculated that joint attention plays a pivotal role for the emergence of language, but some autistic children may acquire language independently of joint attention skills.

Other studies investigating intellectual functioning in MV population have yielded highly heterogeneous results (Bal et al., 2016). It has been reported that, in MV autistic preschoolers, nonverbal intelligence quotient is one of the major predictors of subsequent language gains for those children who acquire some language before the age of 5 (Ellis Weismer and Kover, 2015; Thurm et al., 2007; Thurm et al., 2015; Wodka et al., 2013). Additionally, general intellectual disability has been reported in MV autistic individuals (Hewitt et al., 2012; Luyster et al., 2008; Slušná et al., 2021). However, Bal and colleagues (Bal et al., 2016) reported in 2016 that 16% of children in their sample had non-verbal cognitive abilities within average limits, contrasting with the hypothesis that minimal verbalization is synonymous with cognitive impairment. These results suggest that the mechanisms underlying the lack of language development are not solely attributable to cognitive level, but rather to a heterogeneous set of predictive factors, some of which are precursors to language that vary across the autism spectrum (Pecukonis et al., 2019; Thurm et al., 2007).

As we described, there are several studies that analyze the autistic phenotype in different syndromes (e.g., case reports and syndrome-specific cohorts), but none focus specifically on patients with syndromes that present both autism and are found to be minimally verbal.

Given the notable gap in current literature regarding the clinical and genetic profiles of autistic individuals with minimal verbal abilities, the goal of this study was to assess the prevalence of genetic conditions within MV autistic population and identify genetic features linked to specific syndromes. Furthermore, by analyzing the neuropsychological profiles of a subgroup within this cohort, the study sought to identify clinical phenotypes associated with these genetic factors that is as primary neuropsycological outcome the severity of ASD characteristics and as secondary outcome if children with genetic alterations would exhibit differences in cognitive, adaptive, or behavioural outcomes compared to those without.

We finally hypothesised whether, in the MV autistic population, certain characteristics of the MV profile could be fully explained by the specific genetic condition identified or not.

The novelty of this study is that we tried to apply a systematic approach including both a detailed neuropsychological profile in MV autistic individuals and a in-depth genetic evaluation. Moreover, considering the relatively low prevalence of autism in syndromes, the size of the cohort inclued was significant.

## Materials and methods

### Procedure

Considering the difficulty of identifying whether there are any factors that can help us in better understanding why about 25%–30% of autistic individuals fail to develop functional spoken language, we evaluated and described the clinical and genetic profile of a cohort of 60 ASD pediatric patients who remain minimally verbal past age 5. This study employed a cross-sectional design. Data were retrospectively gathered through a detailed review of the records of patients who attended the Child and Adolescent Neuropsychiatry Unit at a tertiary Children’s Hospital between 2009 and 2022. These patients were referred either by pediatricians due to clinical suspicion of ASD or for follow-up evaluations after receiving an ASD diagnosis. The standard assessment protocol always involved a neuropsychiatric examination, evaluation of cognitive and adaptive functioning, assessment of ASD symptoms, a comprehensive psychopathological assessment and the clinical geneticist evaluation. Children were defined as MV based on the use of module 1 of the ADOS, designed for children aged over 30 months who have no speech or use very few words in simple combinations.

Furthermore all 60 patients underwent several genetic investigations - karyotype, FMR1 (Fragile X Messenger Ribonucleoprotein 1), CMA (Chromosomal Microarray Analysis) and eventually exome sequencing in some selected cases - and were included in an evaluation protocol to identify whether there were any specific clinical or genetic characteristics in the group of MV autistic children. Two different clinical geneticists performed dysmorphology screening on all 60 patients in the cohort. Written informed consent was obtained from parents or legal guardians of each participant included in the study. The study adhered to the principles outlined in the Declaration of Helsinki and was approved by the local Ethics Committee (protocol code: 2423_OPBG_2021, approved on 27 October 2021).

### Participants

According to DSM-5-TR diagnostic criteria, all 60 children and adolescent were diagnosed with ASD.

In particular inclusion criteria included (1) a previous clinical diagnosis of ASD, confirmed by research-reliable staff using the Autism Diagnostic Observational Schedule (ADOS-Generic (ADOS-G), (Lord et al., 2000),) or ADOS Second Edition (ADOS-2) (Lord et al., 2012), Module 1, appropriate for children without phrase speech; (2) grouping was based on the ADOS–2 module completed with the child (minimally verbal = Module 1, based on the use of module 1 designed for children with an absence of language or with production of a few single words (3) chronological age between 5 and 18 years.

Exclusion criteria were age <5 years and presence of organic problems that may cause the phenotype presented by the patient.

### Measures

#### Neuropsychological assessment

##### Autistic symptoms assessment

The diagnosis of ASD was established in accordance with the DSM-5-TR and was confirmed by the administration of the “gold-standard” instruments for the assessment of ASD symptoms, namely, the ADOS-G or ADOS-2 and the Autism Diagnostic Interview-Revised (ADI-R) (Rutter et al., 2003). In our subgroup analysis, only patients with the most recent version of the ADOS (ADOS-2) were evaluated. The ADOS-2 is a semi-structured direct assessment of communication, social interaction, and play or imaginative use of materials for individuals with a suspected diagnosis of ASD. The ADOS-2 consists of five modules designed for children and adults with different levels of language, from nonverbal to verbally fluent; it was administered and scored by licensed neuropsychiatrists. Total score combines symptoms from the Social Affect (SA) and Restricted and Repetitive Behaviors (RRB) domains. In the analyses, the Calibrated Severity Score (CSS) were considered for the ADOS-2. The ADI-R is a standardized, semi-structured interview during which caregivers report information about an individual suspected of having an ASD. The instrument generates algorithm scores for each of the three subdomains of autistic symptoms: qualitative impairments in reciprocal social behavior; qualitative abnormalities in communication and restricted range of interests and/or stereotypic behaviors.

##### Cognitive assessment

Cognitive development was assessed by the Leiter International Performance Scale – 3rd Edition – (Leiter-3) or Leiter international performance scale-revised (Leiter-R) (Roid et al., 2000; Roid and Koch, 2017). The Leiter-3 provides a nonverbal measure of intelligence and assesses the ability to reason by analogy, by matching and perceptual reasoning in general. The Global Nonverbal Intelligent Quotient obtained through this test is based on four subtests: Figure Ground, Form Completion, Classification and Analogies, and Sequential Order. The Leiter-R is a standardized, reliable and valid nonverbal measure of intellectual ability. Four subtests were used to compute the brief intelligence quotient (IQ) score. Two of the four subtests assessed visual perceptual skills (Figure Ground and form completion); the other two assessed fluid reasoning skills (repeated patterns and sequential order). Moreover, we administered the Griffiths Mental Development Scales—Extended Revised (GMDS-ER) when children failed to complete the Leiter-3 (Griffiths et al., 2007). The GMDS-ER provides a measure of development in children aged 0–2 years in five different domains (Locomotor, Personal–Social, Language, Eye and Hand Coordination, and Performance). Every subscale provides a different developmental quotient and a diagnostic indication of problems in early childhood. The average of the quotients of the six subscales provides a Global Developmental Quotient. In the present study, we considered only the nonverbal scores obtained from each instrument, as follows: the Global Nonverbal Intelligence Quotient (NVIQ) of the Leiter-3, brief-IQ of the Leiter-R and the Performance Scale Quotient of the GMDS-ER.

##### Adaptive skills assessment

The ABAS-II (Adaptive Behavior Assessment system - second edition) (Oakland et al., 2011) is a caregiver-compiled adaptive function assessment tool. The instrument investigates several adaptive areas, which can be attributed to three domains:

- Conceptual (Conceptual Adaptive Domain - CAD): Communication, Preschool/School Skills, Self-control;
- Social (Social Adaptive Domain - SAD): Play/Leisure, Socialization;
- Practical (Practical Adaptive Domain - PAD): Self-Care, Home/School Life, Use of Environment, Health and Safety, Work.

There’s also a Global Adaptive Composite (GAC).

Added to these, there is the Motor Skills area, which is limited to the assessment of children from 0 to 5 years old.

#### Behavioral and psychological screening

##### Child behaviour checklist (CBCL)

Behavioral and psychological screening was performed by means of the parent-report questionnaire CBCL (Achenbach et al., 2001). For preschoolers, we used the CBCL for ages 1.5 to 5, which consists of 100 problem items. The instrument generates seven syndrome scales and five DSM-oriented scale profiles, consistent with the diagnostic categories of DSM-IV-TR and DSM-5. For participants aged 6–18 years, we used the CBCL 6–18, which generates eight syndrome and embraces six DSM-Oriented scales. In the current study, we considered the DSM-Oriented scales overlapping in the two versions of the instrument (i.e., versions for ages 1.5-5 and 6–18 years). Clinical cut-off description: T-Scores: 65–69 (Borderline) and 70+ (Clinical) are generated for narrow band scales.

##### Parental stress assessment

To investigate maternal stress levels, the Parenting Stress Index-Short Form (PSI) (Abidin, 1990) was used. PSI is an easy-to administer tool to measure maternal stress. It consists of 36 questions, and each item is rated on a 5-point Likert scale from (1) strongly disagree to (5) strongly agree. The PSI captures three domains—parental distress (PD), parent–child dysfunctional interaction (P-CDI), and difficult child (DC). The sum of all questions results in the Total Stress score. PSI has been translated into several languages and has been frequently used in ASD research (Huang et al., 2014; Leonardi et al., 2021; Papanikolaou et al., 2022; Scibelli et al., 2021).

### Genetic assessment: karyotype, fragile X messenger ribonucleoprotein 1, chromosomal microarray analysis and exome sequencing

Heparin peripheral blood samples were collected for karyotype analysis. GTG-banding was performed according to standard procedures on metaphases obtained from phytohaemagglutinin (PHA)-stimulated lymphocytes. Chromosome analysis was carried out at the 550-band level.

Genomic leukocyte DNA was extracted from Ethylenediaminetetraacetic acid (EDTA) peripheral blood samples of the patients and their parents (when available) by means of Qiagen blood and tissue kit (https://www.qiagen.com), according to the manufacturer’s protocol. DNA quality and quantification were assessed using the NanoDrop® ND-8000 spectrophotometer (Thermo Scientific, United States).

- The Fragile X Messenger Ribonucleoprotein 1 (FMR1) CGG repeats analysis was carried out following the manufacturer’s instruction (AmplideX FMR1 PCR Kit, Asuragen). PCR products were run on 3500 xL Dx Genetic Analyzer (Applied Biosystems).

CMA (Chromosomal Microarray Analysis) was performed using array-CGH or SNP-array platforms:

- Array-CGH was performed using 60K or 180K Agilent Oligo arrays (Agilent Technologies, United States of America) with an effective resolution of 150 kb and 75 kb, respectively. Images were obtained using an Agilent DNA Microarray Scanner and Agilent Scan Control Software while analyses were performed by Agilent CytoGenomics (v 5.3.);
- Single nucleotide polymorphism (SNP)-array analysis was performed using Infinium CytoSNP-850 K BeadChip (Illumina, San Diego, CA). Array scanning data were generated by the iScan system (Illumina), and results were analyzed by the Blue Fuse™ Multi Software Edition 4.5 (release hg38). CNV confirmation and segregation tests on patients and their parents were carried out by real-time PCR using a sybr green assay, as previously described (Livak and Schmittgen, 2001).

DMPK CTG repeats genotyping was obtained using the same methodology trialed in previous studies (van der Plas et al., 2019; Gomes-Pereira et al., 2004). For variant repeat identification, small-pool PCR products underwent AciI enzyme digestion (New England Biolabs United Kingdom Ltd.; restriction site 5′CCGC-3′) and Southern blotting to indicate the presence of CCG interruptions within the CTG repeat array in the expanded allele as previously described (Braida et al., 2010).

Library elaboration and whole exome capture were performed in singleton or in trio by using the Twist Human Core Exome Kit (Twist Bioscience) according to the manufacture’s protocol. The sequencing was carried out on the Illumina NovaSeq 6000 platform. The BaseSpace pipeline (Illumina) and the TGex software (LifeMap Sciences) were employed for the calling- and annotating variants, respectively. Sequencing data were aligned to the hg19 human reference genome and visualized by the Integrative Genome Viewer (IGV).

Next-Generation Sequencing (NGS) variants and Copy Number Variation (CNV) identified by SNParray and array-CGH were classified according to the ACMG/AMP criteria (Richards et al., 2015) and the ACMG/ClinGen recommendations (Riggs et al., 2021).

As previously mentioned, there is variability in the terminology used to describe the genetic cause of an autistic phenotype (Vorstman and Scherer, 2023).

The term syndrome has been widely used related to ASD associated with genetic conditions; the construct is understood by some to imply the presence of additional symptoms,the latter sometimes specified as “neurological disorders,” “dysmorphic features typically associated with genetic variants,” or as “major anomalies, suggesting malformation.” Although all these definitions can, with some goodwill, be grouped as “ASD with additional phenotypes,” another usage of the concept syndromic autism hinges on the presumed genetic etiology as its core defining feature. Here, the concept is predicated on the presence, or presumed presence, of an identifiable genetic disorder in individuals with ASD or significant autistic traits. Hence the hypothesis in recent literature of a new nomenclature: using “with genetic causative alterations” for individuals in whom a pathogenic variant is thought to be causative of or contribute to the ASD phenotype and “without genetic causative alterations” for whom there are not pathogenic variants (Vorstman and Scherer, 2023). In this study we preferred using the latter terminology.

## Results

### Genetic assessment

Noteworthy, in this cohort of 60 ASD individuals who remain *minimally verbal* past age 5, a clear genetic association was disclosed in 14 cases. In particular they were identified: one case of 1q21.1 duplication syndrome (OMIM #612475); a patient with Greig cephalopolysyndactyly syndrome (OMIM #175700); a Kleefstra syndrome case (OMIM #610253); a case of macrocephaly/autism syndrome (OMIM #605309) due to a pathogenic variant in *PTEN*; a Costello syndrome patient (OMIM #218040); a boy carrying the 16p11.2 microdeletion syndrome (OMIM #611913); a girl with tuberous sclerosis 2 (OMIM #613254); one case of Intellectual developmental disorder with hypotonia, impaired speech, and dysmorphic facies (OMIM #619556) due to a heterozygous variant in *TNPO2*; a case of myotonic dystrophy (OMIM #160900); two cases of Phelan McDermid syndrome (OMIM #606232), one due to the 22q13.33 microdeletion and one due to a heterozygous pathogenic variant in *SHANK3*; a boy with a 18q22.3–18q23 deletion, syndrome (Tassano et al., 2016); one patient with Down syndrome; and lastly the very rare case of a patient with Congenital anomalies of kidney and urinary tract syndrome with or without hearing loss, abnormal ears, or developmental delay (CAKUTHED) (OMIM #617641), due to heterozygous variant in PBX1, and Intellectual developmental disorder with autism and speech delay (OMIM # 606053), associated to a heterozygous pathogenic variation in *TBR1*. The patients’ sex, the age at diagnosis, the specific pathogenic variations of each case, the genes involved, segregation studies, inheritance pattern, and a short description of every identified disorder are reported in Table 1, together with the estimated autism frequency for each disorder, when available.

**TABLE 1:** Genetic alterations detected in 14 MV autistic individuals.

Case	M/F	Age at diagnosis	Patient’s variation	OMIM morbid genes involved	Syndrome	Short clinical description of the disorder	Segregation	Inheritance pattern	ACMG classification	Estimated ASD frequency when available	References
1	M	18 ys old	arr [GRCh37] 1q21.1q21.2 (146506310_147824207)x3	GJA5, GJA8	1q21.1 duplication syndrome (OMIM #612475)	Chromosome 1q21.1 duplication syndrome include a broad range of signs and symptoms as macrocephaly, mild to moderate developmental delay and learning difficulties, ASD or autistic-like behavior, heart anomalies, seizures, dismorphic features. It tipically displays reduced penetrance and variable expressivity	Adopted	AD *(de novo* or transmitted)	LP	35.6% for ASD or autistic features and 8.5% for seizures	Bourgois et al. (2024); Dolcetti et al. (2013)
2	M	1 year and 2 months	*GLI3* heterozygous c.3677del (p.Pro1226GInfs*4)	*GLI3*	Greig cephalopolysyndactyly syndrome (GCPS; OMIM #175700)	GCPS is characterized by macrocephaly, widely spaced eyes associated with increased interpupillary distance, preaxial polydactyly with or without postaxial polydactyly, and cutaneous syndactyly. Developmental delay, intellectual disability or seizures are uncommon (<10%) and may be more frequent in individuals with large deletions (>300 kb) encompassing GLI3. Approximately 20% of individuals with te disorder have hypoplasia or agenesis of the corpus callosum	Pat	AD	P	Sporadic	Biesecker and Johnston (2001); Siracusano et al. (2019)
3	F	7 ys old	arr [GRCh37] 9q34 (140414410–140633931)x1	EHMT1	Kleefstra syndrome (KLEFS1; OMIM #610253)	KLEFS1 is a neurodevelopmental disorder characterized by intellectual disability (moderate-to-severe), autistic-like features, childhood hypotonia and distinctive facial features. Few individuals have mild delay and total IQ within low-normal range. Most patients have severe expressive speech delay with little speech development. Nonverbal communication is possible. Other findings include heart defects, renal/urologic defects, genital defects in males, severe respiratory infections, epilepsy/febrile seizures, psychiatric disorders, and extreme apathy or catatonic-like features after puberty	*de novo*	AD (ususally *de novo*)	P	Around 95.7% of kleefstra patients are diagnosed with ASD	Kleefstra and de Leeuw (2010); Vermeulen et al. (2017)
4	F	14 ys old	*PTEN* heterozygous c.697C>T (p.Arg233Ter)	PTEN	Macrocephaly/autism syndrome (OMIM #605309)	PTEN hamartoma tumor syndrome (PHTS) is associated with a relatively high prevalence of ASD. PHTS results in a multitude of presentations that range from increased cancer risks to macrocephaly and neurodevelopmental impairment. Noteworthy, up to 20% of individuals with ASD with macrocephaly may have a pathogenic PTEN variant	No mat (father not available)	A	P	Up to 20% of children of ASD cases	Busch et al. (2019)
5	M	3 ys old	*HRAS* eterozygous c.108_110dupAGA (p.E37dup) (*de novo*)	HRAS	Costello syndrome (OMIM #218040)	Costello syndrome shows a wide phenotypic spectrum ranging from a mild to a severe phenotype with early-lethal complications. The syndrome is characterized by severe postnatal feeding difficulties, short stature, developmental delay or intellectual disability; coarse facial features, curly or sparse, fine hair, loose, soft skin with deep palmar and plantar creases, papillomata of the face and perianal region; diffuse hypotonia and joint laxity with ulnar deviation of the wrists and fingers; tight achilles tendons; cardiac hypertrophy, congenital heart defects and arrhythmia. Relative or absolute macrocephaly is present; postnatal cerebellar overgrowth can result in the development of a chiari I malformation with associated anomalies including hydrocephalus or syringomyelia. Individuals with costello syndrome have an approximately 15% lifetime risk for malignant tumors including rhabdomyosarcoma and neuroblastoma in young children and transitional cell carcinoma of the bladder in adolescents and young adults	*de novo*	AD	P	About 44% with costello syndrome have a clinical diagnosis of ASD	Gripp and Weaver (2023); Adviento et al. (2014); Alfieri et al. (2014)
6	M	5 ys old	arr [GRCh37] 16p11.2 (29656684_30190568)x1	ALDOA KIF22 PRRT2 TBX6 TLCD3B	16p11.2 microdeletion syndrome (OMIM #611913)	Chromosome 16p11.2 deletion syndrome is characterized by motor speech and language disorder, motor coordination difficulties, psychiatric conditions, and autistic features. Most of the individuals experience some degree of developmental delay. Many affected individuals do not have intellectual disability (with an IQ < 70), but may show below average cognition and learning disabilities in both verbal and nonverbal domains. Obesity is a feature of the disorder and generally emerges in childhood. Seizures are observed in approximately 25% of individuals with the deletion. Vertebral anomalies, hearing impairment, macrocephaly, and cardiovascular malformation have been observed	Mat	AD	LP	Up to 1% of all ASD cases	Taylor et al. (2021); Fernandez et al. (2010)
7	F	1 year old	*TSC2* heterozygous c.4663-7_4663-1delCCCACAG, intron 35	TSC2	Tuberous sclerosis 2 (TSC2; OMIM #613254)	TSC2 involves abnormalities of the skin (hypomelanotic macules, confetti skin lesions, facial angiofibromas, shagreen patches, fibrous cephalic plaques, ungual fibromas), brain (subependymal nodules, cortical tubers, and subependymal giant cell astrocytomas, seizures, associated neuropsychiatric disorder), kidneys (benign renal angiomyolipomas, epithelial cysts, oncocytoma, renal cell carcinoma), heart (rhabdomyomas, arrhythmias) and lungs (lymphangioleiomyomatosis, multifocal micronodular pneumonocyte hyperplasia). Central nervous system-related problems are the leading cause of morbidity, whereas kidney disease is the leading cause of mortality	Not known	AD	P	About 25% of individuals with tuberous sclerosis have autism and 40%–50% meet diagnostic criteria within the ASD	Northrup et al. (1999); Smalley (1998)
8	M	1 year old	arr [GRCh37] 18q22.3q23 (72376739_78015180)x1	CTDP1, TSHZ1, TXNL4A, ZNF407	18q22.3–18q23 deletion syndrome	Overlapping 18q22.3–18q23 deletion has been reported in a 16-month-old male infant showing delayed motor development, peculiar neuroradiological abnormalities, growth retardation, global hypotonia, hyperlaxity, hyporeflexia and dismorphic features	*de novo*	Sporadic (AD)	P	Unknown	Tassano et al. (2016)
9	M	14 ys old	*TNPO2* heterozygous c.926C>T (p.Ser309Leu)	*TNPO2*	Intellectual developmental disorder with hypotonia, impaired speech, and dysmorphic facies (IDDHISD; OMIM #619556)	IDDHISD is characterized by global developmental delay with impaired intellectual development and poor or absent speech, hypotonia, ophthalmologic abnormalities and nonspecific dysmorphic features. Some affected individuals have seizures and a few have involvement of other organ systems	*de novo*	AD	P	Unknown	Goodman et al. (2021)
10	M	15 ys old	*DMPK* heterozygous 400–500 CTG	DMPK	Steinert disease/myotonic dystrophy 1 (DM1; OMIM #160900)	DM1 is a multisystemic disorder affecting both skeletal and smooth muscle, eye, heart, endocrine system, and central nervous system. The clinical picture, ranging from mild to severe, is characterized by hypotonia, severe generalized weakness at birth, respiratory insufficiency and early death; intellectual disability is common. DM1 is caused by the expansion of a CTG trinucleotide repeat in the noncoding region of DMPK. CTG repeat length exceeding 34 repeats is abnormal	​	AD	P	36% of a cohort containing congenital and juvenile-onset DM1	Bird (1999); Angeard et al. (2018)
11	F	19 ys old	arr [GRCh37] 22q13.33 (51126986_51195728)x1 dn	*SHANK3*	Phelan McDermid syndrome (PMS; OMIM #606232)	PMS is a neurodevelopmental disorder with variable features. Common features include neonatal hypotonia, global developmental delay, normal to accelerated growth, absent to severely delayed speech, autistic behavior, and minor dysmorphic features. Some individuals experience regression/loss of skills, epilepsy, abnormal gait, and sleep disturbance. It can be due to single nucleotide pathogenetic variants in SHANK3 gene or to the deletion of the 22q11.33 genomic region, incuding at least part of the gene	*de novo*	AD	P	About 53% of individuals with PMS had a clinical diagnosis of ASD	Prasad et al. (2000); Phelan et al. (2005); Oberman et al. (2015)
12	M	16 ys old	*SHANK3* heterozygous c.4818_4819insA (p.Glu1608fs*71)	*SHANK3*			*de novo*		P	About 53% of individuals with PMS had a clinical diagnosis of ASD	
13	M	1 year old	XY+21	​	Down syndrome	Down syndrome (trisomy 21) is a genetic disorder caused by the presence of all or a portion of a third chromosome 21. Patients typically present with mild to moderate intellectual disability, growth retardation, and characteristic facial features. Further clinical signs are congenital heart defects, gastrointestinal abnormalities, epilepsy and brain malformatons	​	AD	P	5%–39%	Akhtar and Bokhari (2025); Lowenthal et al. (2007); Moss et al. (2013)
14	M	15 ys old	*PBX1* heterozygous c.121C>T (p.Gln41Ter) *TBR1* heterozygous c.1896C>G (p.Tyr632Ter)	PBX1 TBR1	Congenital anomalies of kidney and urinary tract syndrome with or without hearing loss, abnormal ears, or developmental delay (CAKUTHED; OMIM #617641) Intellectual developmental disorder with autism and speech delay (IDDAS; OMIM # 606053)	CAKUTHED is a highly pleiotropic developmental disorder characterized by variable congenital anomalies of the kidney and urinary tract, sometimes resulting in renal dysfunction or failure, peculiar facial features, and abnormalities of the outer ear, often with hearing loss. Most patients have global developmental delay IDDAS is a neurodevelopmental disorder characterized by varying degrees of intellectual disability, ASD and language deficits. TBR1 interacts with FOXP2, a transcription factor implicated in speech/language disorders, and this interaction is disrupted by pathogenic mutations affecting TBR1	*de novo* Mat	AD AD	P LP	Unknown	Slavotinek et al. (2017) Deriziotis et al. (2014)

P, pathogenetic; LP, likely pathogenic; AD, autosomal dominat.

The percentage of genetic disorders detected in the present series is 22.6%.

### Neuropsychological assessment

#### Children and adolescents without an associated genetic cause

Out of 46 patients, 39 were male and 7 were female - M age 8.76, Standard Deviation (SD) 2.44; mean nonverbal IQ 58.68.

We have neuropsychological data from approximately 32 children and adolescents (Tables 2, 3). Among these, 96.6% had an ADOS-2 total score above 12, meeting the clinical cut-off for autism. Intellectual functioning assessments indicated that 77.5% of the children had an NVIQ score below 70. Specifically, 15% of the children had mean NVIQ of 30, 15% had mean NVIQ of 45, and 47.5% had mild mean NVIQ score of 69. Regarding adaptive functioning, 92.8% had a Global Adaptive Composite (GAC) below 70 on the ABAS-II, while 96.4% scored below 70 on the Daily Living Skills scale (CAD), 89.2% on the Social Adaptive Domain (SAD), and 89.2% on the Practical Adaptive Domain (PAD), all reflecting significant adaptive impairments. Behavioral assessments showed that 35.6% of children exhibited clinically significant internalizing problems (CBCL score >63), 25.8% demonstrated clinically significant externalizing problems, and 38.8% had total problem scores within the clinical range. Additionally, 46.6% of parents reported a parental stress score above 90, indicating clinically significant levels of stress.

**TABLE 2:** ADOS-2 group comparisons (without vs. with genetic causative alterations). Reported: N, mean (SD), Welch’s t (df, two-tailed p), Bonferroni-adjusted p (m = 4; α = 0.0125), Cohen’s d [95% CI], and bootstrap d [95% CI] (10,000 within-group resamples).

Measure	Group without-genetic causative alterations mean (SD), n	Group with genetic causative alterations, mean (SD), n	t (df)	p	P (bonf)	Cohen’s d [95%CI]	Bootstrap d [95%CI]
ADOS-2 TOT	19.66 (4.58) n = 32	15.38 (5.09) n = 8	2.16 (10.02)	0.056	0.224	0.91 [0.11, 1.72]	0.91 [0.19, 1.82]
ADOS-2 SA	14.29 (3.74) n = 31	10.75 (3.91) n = 8	2.29 (10.54)	0.044	0.175	0.94 [0.13, 1.74]	0.94 [0.22, 1.93]
ADOS-2 RRB	5.19 (1.37) n = 32	4.63 (1.68) n = 8	0.87 (9.47)	0.406	1.000	0.39 [-0.39, 1.17]	0.40 [-0.43, 1.38]
ADOS-2 TOT CSS	7.10 (1.16) n = 31	5.50 (1.30) n = 8	3.14 (10.05)	0.010*	0.042*	1.35 [0.51, 2.18]	1.36 [0.66, 2.28]

Abbreviations: SA, social affect; RRB, restricted and repetitive behaviors; CSS, calibrated severity score.

**TABLE 3:** Group comparisons on IQ; ABAS-II (GAC, CAD, SAD, PAD); CBCL (INT, EXT, TOT); PSI Total between participants without vs. with genetic causative alterations. Reported: mean (SD), n per group, Welch’s t (df, two-tailed p), Bonferroni-adjusted p, Cohen’s d [95% CI], and bootstrap d [95% CI] (10,000 within-group resamples).

Measure	Group without-genetic causative alterations mean (SD), n	Group with genetic causative alterations, mean (SD), n	t (df)	p	P (bonf)	Cohen’s d [95%CI]	Bootstrap d [95%CI]
IQ	58.68 (17.42) n = 40	57.11 (15.52) n = 9	0.26 (12.95)	0.799	1.000	0.09 [-0.63, 0.82]	0.09 [-0.59, 0.79]
ABAS II GAC	49.79 (13.07) n = 28	43.88 (7.58) n = 8	1.62 (20.15)	0.121	1.000	0.49 [-0.31, 1.28]	0.53 [-0.13, 1.08]
ABAS II CAD	54.71 (10.62) n = 28	52.25 (6.58) n = 8	0.80 (18.61)	0.434	1.000	0.25 [-0.54, 1.04]	0.28 [-0.53, 0.80]
ABAS II SAD	57.79 (11.18). n = 28	56.88 (4.08) n = 8	0.35 (31.55)	0.724	1.000	0.09 [-0.70, 0.88]	0.08 [-0.50, 0.53]
ABAS II PAD	50.11 (15.05) n = 28	44.50 (11.19) n = 8	1.15 (15.05)	0.268	1.000	0.39 [-0.40, 1.18]	0.43 [-0.34, 0.93]
CBCL INT	57.48 (8.83) n = 31	59 (11.32) n = 8	−0.35 (9.31)	0.734	1.000	−0.16 [-0.94, 0.62]	−0.19 [-1.12, 0.70]
CBCL EXT	57.58 (6.77) n = 31	58.13 (11.45) n = 8	−0.12 (8.30)	0.907	1.000	−0.07 [-0.85, 0.71]	−0.07 [-1.12, 1.01]
CBCL TOT	61.45 (7.87) n = 31	61.88 (11.12) n = 8	−0.10 (8.89)	0.923	1.000	−0.05 [-0.83, 0.73]	−0.06 [-1.01, 0.91]
PSI TOT	88.04 (21.51) n = 28	83.88 (24.52) n = 8	0.43 (10.29)	0.673	1.000	0.19 [-0.60, 0.97]	0.21 [-0.63, 1.08]

Abbreviations: IQ, intelligence quotient; ABAS-II, GAC, general adaptive composite; CAD, conceptual; SAD, social; PAD, practical; CBCL:INT, internalizing; EXT, externalizing; TOT, total; PSI TOT, Parenting Stress Index—Total.

#### Children and adolescents with an associated genetic cause

Out of 14 patients, 10 were male and 4 were female (M age 9.37, SD 5.86; mean nonverbal IQ 57.11). We have neuropsychological data from approximately 8 children. Among these, 87.5% had an ADOS-2 total score above 12, reaching the clinical threshold for autism. Intellectual assessments revealed that 77.7% of the children had an NVIQ score below 70. Specifically, 11.1% of the children had mean NVIQ of 32, 33.3% had mean NVIQ of 45, and 33.3% had mean NVIQ of 66. Adaptive functioning assessments indicated that all syndromic children (100%) had a GAC score below 70 on the ABAS-II, as well as subscale scores below 70 for Daily Living Skills (CAD), Social Skills (SAD), and Practical Skills (PAD), demonstrating pervasive adaptive deficits. Regarding behavioral difficulties, 62.5% of children exhibited clinically significant internalizing problems (CBCL score >63), 37.5% had clinically significant externalizing problems, and 50% had total problem scores in the clinical range. Additionally, 37.5% of parents reported a parental stress score above 90, reflecting clinically significant stress levels.

#### Children and adolescents without versus with genetic cause associated

**FIGURE 1:**
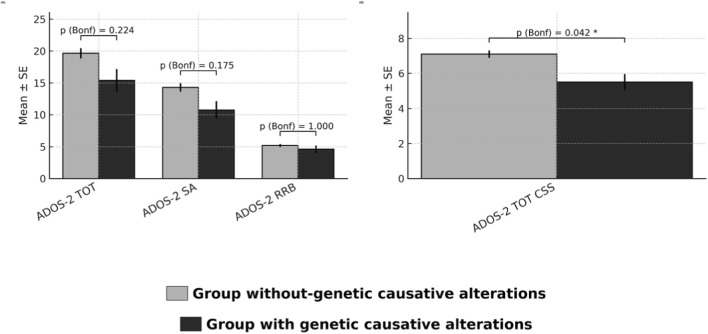
ADOS-2 outcomes by genetic status. **(A)** Algorithm scores: Total (TOT), Social Affect (SA), Restricted and Repetitive Behaviors (RRB). **(B)** Calibrated Severity Score (CSS; range 1–10). Bars show mean ± SE. Grey = participants without genetic causative alterations; black = participants with genetic causative alterations. Labels report Bonferroni-adjusted p from two-tailed Welch’s t; stars indicate significance (*p* < 0.05 = *, *p* < 0.01 = **, *p* < 0.001 = ***). Multiplicity controlled within the ADOS-2 group (m = 4; α = 0.0125).

All data were uploaded to SPSS (Field, A. *Discovering Statistics Using IBM SPSS Statistics*; Sage: Thousand Oaks, CA, United States, 2013) version 27 for analysis. We compared participants *without versus* with genetic causative alterations on ADOS-2 domains (SA, RRB, ADOS TOT, ADOS TOT CSS) and on secondary outcomes (IQ, ABAS-II GAC/CAD/SAD/PAD, CBCL INT/EXT/TOT, PSI Total). For each continuous measure we used two-tailed Welch’s *t* (to accommodate unequal variances and unbalanced group sizes; *Ns* vary by measure due to missing data). To control multiplicity, we applied Bonferroni within two *a priori* families (ADOS-2: *m* = 4, α = 0.0125; other measures: *m* = 9, α = 0.0056). We reported Cohen’s *d* with 95% CIs and nonparametric bootstrap estimates of *d* with 95% CIs (10,000 within-group resamples). Full descriptive statistics and inferential results are provided in Table 2 (ADOS-2) and Table 3 (IQ, ABAS-II, CBCL, and PSI). Within ADOS-2 outcomes, the genetic subgroup showed lower severity on average; however, only ADOS-2 TOT CSS remained significant after multiplicity control (*t* (10.05) = 3.14, *p* = 0.010, Bonferroni *p* = 0.042; *d* = 1.35 [0.51, 2.18]; bootstrap *d* = 1.36 [0.66, 2.28]). ADOS-2 TOT and Social Affect were nominally significant with large effect sizes (both *d* ≈ 0.9) but did not survive Bonferroni; Restricted Repetitive Behavioral scale showed no evidence of group difference (Figure 1 shows group comparison on ADOS-2).

**FIGURE 2:**
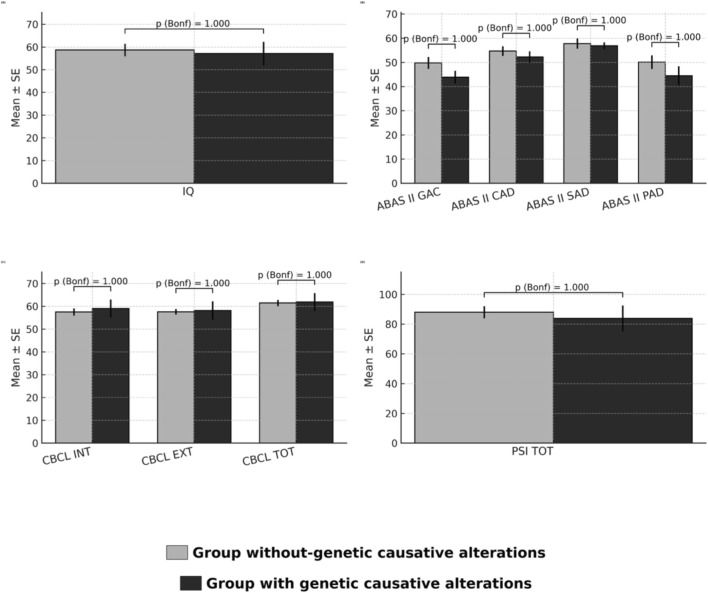
Group comparisons on secondary outcomes. **(A)** IQ; **(B)** ABAS-II (GAC, CAD, SAD, PAD); **(C)** CBCL (INT, EXT, TOT); **(D)** PSI Total. Bars show mean ± SE. Grey = participants without genetic causative alterations; black = participants with genetic causative alterations. Labels report Bonferroni-adjusted p from two-tailed Welch’s t; stars denote significance (*p* < 0.05 = *, *p* < 0.01 = **, *p* < 0.001 = ***). Multiplicity controlled within the secondary-outcome group (m = 9; α = 0.0056).

Across cognitive functioning (IQ), Adaptive functioning (ABAS-II), emotional and behavioral functioning (CBCL), and parental stress (PSI), no comparison survived Bonferroni, and effect sizes were small to moderate with 95% CIs typically spanning zero. Given the small genetic subgroup (*n* = 8), we interpret all non-significant trends cautiously (Figure 2 shows group comparison on secondary outcomes). See Tables 2, 3 for complete results.

## Discussion

The first aim of this study was to conduct a genetic analysis of a large sample of autistic children and adolescents with MV characteristics. The finding was a 22.6% prevalence of genetic disorders, characterized by a diverse distribution of duplication and deletion and variations patterns affecting various gene loci, each associated with distinct clinical phenotypes, as detailed in Table 1.

To our knowledge, the data available in the literature indicate that approximately 10% of all autistic individuals typically exhibit dysmorphic traits and/or malformations and are classified as having syndromic autism (Zafeiriou et al., 2013; Styles et al., 2020). Therefore, in our sample, the prevalence of genetic syndromes was found to be twice as high as in the general autistic population.

“Syndromic” autism includes deletions and duplications syndromes (e.g., 1q24.2, 15q11-q13, 16p11.2, 22q11), chromosomal aneuploidies (e.g., Down syndrome) and monogenic syndromes (e.g., Fragile X syndrome, Angelman syndrome, Rett syndrome, Tuberous Sclerosis Complex - TSC, Neurofibromatosis and PTEN hamartoma tumor syndrome) (Ziats et al., 2021; Genovese and Butler, 2023).

Some genetic syndromes have been reported to have higher autism prevalence rates, more similar to those observed in our MV cohort: for Kleefstra Syndrome the prevalence of behavioral disorders, such as autism spectrum disorder, is about 38% (Frazier et al., 2025); in Myotonic Dystrophy type 1 the prevalence of ASD was 14%, with congenital onset being 79% more common than juvenile onset (Pascual-Morena et al., 2024); the reported prevalence of ASD in Down syndrome is 16% (Santoro et al., 2021). Adviento and colleagues found in four major RASopathies (neurofibromatosis type 1 - NF1, Noonan syndrome - NS, Costello syndrome - CS, and cardio-facio-cutaneous syndrome - CFC) a high prevalence of ASD traits using Social Communication Questionnaire (SCQ) (85% scored at the threshold of 15 or higher) (Adviento et al., 2014).

For other genetic conditions identified in this study, the prevalence of them in the autistic population is known: about 10%–20% of autistic individuals and macrocephaly have germline PTEN mutations (Yehia et al., 2020).

It is important to note that the overall prevalence of ASD within the syndrome has been found to be significantly higher than the prevalence of the syndrome among ASD cases (Styles et al., 2020). Indeed, for the best characterized syndromes, we are aware of both the prevalence of the syndrome in autistic individuals and the prevalence of ASD in the syndrome: for TSC different studies have shown that 3%–4% of autistic subjects may have TSC; conversely, studies of TSC patients have identified much higher rates of autism, ranging from 17% to 61% (Curatolo et al., 2004). For Phelan-McDermid syndrome (PMS) approximately 1% of individuals with autism are estimated to have SHANK3 mutations while up to 84% of individuals with PMS met criteria for ASD (Frank, 2021).

For 1q21.1 duplication syndrome (Xavier et al., 2018), Greig cephalopolysyndactyly syndrome (Siracusano et al., 2019) and Intellectual developmental disorder with autism and speech delay associated to a heterozygous pathogenic variation in *TBR1* (Cao et al., 2021) we do not have prevalence data regarding ASD but merely case reports.

For intellectual developmental disorder with hypotonia, impaired speech, and dysmorphic facies due to a heterozygous variant in *TNPO2* (Goodman et al., 2021) and CAKUTHED due to heterozygous variant in PBX1 (Alankarage et al., 2020) there are only functional studies.

To our knowledge, none of the cited studies analyze the prevalence of MV autistic individuals in the genetic syndromes studied, athough there is evidence of a gap in the literature concerning the exploration of factors that may affect the language development in autistic individuals (Vogindroukas et al., 2022) and the necessity of examining large cohorts of autistic individuals with MV traits (Koegel et al., 2019). It is broadly acknowledged that an underlying etiological diagnosis, mainly through genetic testing, particularly for autistic children and adolescents with severe symptoms and notable language impairments, is a critical prerequisite for comprehensive management (Hyman et al., 2020). This underscores the significance, in clinical practice, of performing in-depth genetic screening in MV autistic individuals to detect a syndrome that could influence prognostic outcomes for patient care (for instance, consider the cancer surveillance guidelines for individuals with PTEN hamartoma tumor syndrome) (Tischkowitz et al., 2020).

Going now to analyze the neuropsychological profile in a subgroup of patients, our study identified an interesting second finding: the total raw ADOS-2 scores of MV subjects with genetic causative alterations were less severe than those of MV individuals without genetic causative alterations, in a total population that nevertheless by characteristics has high ADOS scores (mean of ADOS-2 total raw scores: 15.38 in MV autistic group with genetic causative alterations *versus* 19.66 in MV autistic group without genetic causative alterations). It has already been described in the literature that the clinical syndromic presentations of autism with genetic causative alterations are extremely heterogeneous, which could be attributed to differences in genetic background, eventual epigenetic influences, and to the distinct characteristics of the population (Styles et al., 2020). With respect to the characteristics of the population we studied: there are no studies in the literature specifically describing the autistic phenotype in syndromic MV individuals. The information on the severity of ASD (e.g., using ADOS-2 Module 1) and language impairment (which we can trace back to a MV autistic population) comes indirectly from case reports analyzing autistic functioning in some of the more studied syndromes. In a recent study (Barone et al., 2023), the autistic phenotype profile was analyzed in two siblings with PARK2 microdeletion as copy number variations (CNVs) involving PARK2 have been associated with ASD. This study pointed out that the younger sibling had minimally verbal autism and severe intellectual disability, whereas his older brother presented high-functioning autism and preserved speech. This is consistent with the hypothesis indicating that polygenic effects acting in summation may explain the heritability and clinical heterogeneity of ASD.

A critical point concerns the interpretation of ADOS-2 severity scores. The finding that MV children without genetic causative alteration exhibited greater autism symptom severity may in part reflect differences in expressive language abilities rather than true differences in core autistic features. Several studies have noted that ADOS calibrated severity scores, particularly in Modules 1 and 2, are influenced by verbal ability, with lower levels of spoken language associated with higher severity ratings (Hus et al., 2014; Gotham et al., 2009). Thus, it is possible that the apparently greater severity observed in the group without genetic causative alteration partly reflects their language profile rather than a genuine difference in autism symptomatology.

One of the most studied syndromes is the Phelan-McDermid Syndrome, which as we have already reported, is associated with ASD but also with Intellectual Disability (ID) and speech and language impairment (absent to severely delayed speech) which are present in all cases (Frank, 2021). Considering Down Syndrome (DS) ad ASD, Godfrey and colleagues in 2019 compared children with co-occurring ASD + DS *versus* children with DS *versus* cognitively matched ASD children (Godfrey et al., 2019). The authors found fewer global ASD symptoms on the ADI-R for autistic children with DS compared to matched autistic children. However, autistic children with DS had more evenly distributed social communication and restricted and repetitive behavior scores while autistic children only had more severe SC than RRB scores. Another study examining domain scores on ADOS Module 1 by Oxelgren et al. (2019). Similarly found lower communication scores for the ASD + DS group compared to an idiopathic ASD only group: autistic children with DS showed significantly lower scores on stereotyped use of phrases (Oxelgren et al., 2019). To explain these findings, Godfrey M. and colleagues thought that it might be related to the ASD + DS group’s low verbal abilities (Godfrey et al., 2019). Indeed, since most observational measures of ASD symptom severity are strongly influenced by language level (Anderson et al., 2007; Schaeffer et al., 2023), it is still necessary to understand whether there is a possible association between the severity of core symptoms and language abilities. Our hypothesis is that, in the MV autistic population without genetic causative alteration, there are specific and unknown characteristics of the MV profile (absence of language development beyond 5 years of age) which have a greater impact than the individual genetic condition reported but this is still speculative and requires longitudinal validation.

We hypothesize that, in MV autistic individuals without a genetic causative alteration, specific and yet unknown features of the MV phenotype may have a greater influence on clinical presentation than the presence of a known genetic condition. For instance, it is possible that, in the absence of a single high-impact genetic variant, the cumulative effect of multiple genetic and neurodevelopmental factors, together with early language deficits, contributes to the more severe presentation observed in this subgroup. However, this interpretation remains speculative and requires validation through longitudinal and multi-omic studies aimed at disentangling the respective contributions of language, cognition, and genetics to the MV phenotype.

Our finding that minimally verbal children without a known genetic condition showed greater autism severity than those with genetic causative alterations requires careful contextualization. Previous studies comparing idiopathic and syndromic ASD have reported mixed results, with some evidence suggesting that syndromic cases present with broader developmental impairments, while idiopathic ASD may show relatively greater severity in core social-communication domains (Oxelgren et al., 2019; Thurm et al., 2019). One possible explanation, as we already told, is that ADOS-2 severity scores may be disproportionately influenced by expressive language abilities (Gotham et al., 2009), which are often more profoundly impaired in MV children without genetic causative alteration. However, alternative mechanisms should also be considered. It is plausible that children without genetic causative alteration may carry a higher polygenic risk load for ASD, in line with recent genomic studies highlighting cumulative contributions of common variants to autism severity (Weiner et al., 2017). Epigenetic mechanisms, such as environmentally mediated changes in gene expression, may also modulate phenotypic severity independently of rare, highly penetrant variants (Loke et al., 2015). Finally, the possibility of undetected genetic variants—including non-coding mutations, structural rearrangements, or low-level mosaicism not captured by standard diagnostic pipelines—cannot be excluded.

Taken together, these considerations suggest that the apparently greater severity in MV children without genetic causative alteration may reflect not only assessment bias linked to language profile, but also a combination of polygenic, epigenetic, and unidentified genetic contributions. This highlights the need for comprehensive genomic testing (e.g., whole-genome sequencing, epigenomic profiling) in future studies of MV populations to disentangle the relative roles of rare syndromic mutations versus broader genetic and environmental liabilities in shaping the clinical phenotype.

Therefore, future studies should analyze the genetic profile of large cohorts of MV autistic children and adolescents to evaluate the possible association with the neuropsychological profile and to better characterize a specific phenotype. Furthermore, when comparing non-verbal intellectual and adaptive functioning, as well as emotional and behavioral problems, no significant differences emerged between the group of MV autistic children and adolescents with associated genetic causes and those without them. According to the literature, most of the syndromic conditions identified in our population are more frequently linked to lower intellectual and adaptive functioning (Samanta, 2020; Xavier et al., 2018; Bradbury et al., 2022; Frank, 2021; see Table 1) compared to the general population. This effect is further amplified in ASD populations when a genetic syndrome (e.g., Down syndrome) is present (Thurm et al., 2019). However, it is important to note that our entire cohort of children exhibits an average NVIQ below 70. As previously reported, general intellectual disability has been described in MV autistic individuals (Hewitt et al., 2012; Luyster et al., 2008; Slušná et al., 2021), yet considerable heterogeneity has been observed, highlighting that language impairment cannot be fully explained by cognitive difficulties (Table 1). It is likely that in our sample, differences between the two groups are not apparent due to the relatively homogeneous cognitive and adaptive functioning of the population studied. Once again, these findings suggest that MV characteristics may play a significant role in our cohort of patients. Additionally, although few studies have examined how emotional and behavioral problems change in children with different language abilities (Fok and Bal, 2019), some psychiatric comorbidities (e.g., anxiety, depression) have been shown to manifest differently in individuals with varied intellectual and language abilities (Einfeld and Aman, 1995). Therefore, the lack of differences in emotional and behavioral profiles, regardless of the presence of associated genetic causes, may be attributed to our sample’s inclusion of individuals who either did not develop language or produced only a few words beyond the age of six.

This study has limitations that should be considered: the limited neuropsychological assessments conducted on a reduced subset of patients rather than the entire sample (given the lack of availability of complete neuropsychological assessments for the entire sample), the retrospective nature of the analyses, and the classification of MV. Consistent with previously selected samples (Guerrera et al., 2025), our sample was classified as minimally verbal (MV) according to one of the definitions reported in the literature, based on both linguistic functioning and age. However, given the absence of a universally established operational definition of MV in autistic individuals, the findings of the present study cannot be considered fully comparable to those of previous research. A similar caveat applies to the assessment tools employed to define the sample. Although we used Module 1 of the ADOS, which is recognized in the literature as valid under certain definitions, variability in the instruments adopted across studies further limits direct comparability. This variability reflects both differences in research aims and the lack of consensus on a universally accepted threshold. In future studies a more precise and consistent operational definition of MV would therefore enhance comparability, facilitate meta-analytic synthesis, and improve translation of research into clinical practice.

Furthermore, although the CBCL is among the most widely used tools for behavioral and psychological screening in autistic children, it includes numerous items that are directly influenced by verbal language skills, which may not be entirely appropriate for autistic and MV individuals (DiStefano et al., 2016). Another limitation of our study lies in its retrospective design, which resulted in heterogeneity in the genetic testing performed across participants.

While all participants underwent first-tier genetic testing (karyotype, FMR1, and CMA), only two underwent exome sequencing, based on clinical evaluation and the presence of peculiar somatic features or syndromic features. Consequently, a large group of individuals negative classified as “without genetic causative alteration” may in fact carry a yet undetected pathogenic variant. This methodological limitation should be addressed in future prospective studies through a more systematic use of uniform, high-resolution genomic testing (e.g., WES or WGS) for all participants in order to enhance the diagnosis rate and to allow a more accurate genotype–phenotype characterization.

Moreover, future research should focus on the need for uniform testing protocols in order to allow more robust genotype–phenotype correlations.

Nevertheless, to our knowledge, this is the first study to investigate genetic causes and neuropsychological characteristics in a relatively large sample of MV autistic children and adolescents and to define the neuropsychological profile within this population. It examines the prevalence of genetic syndromes associated with MV autistic traits and compares the clinical profile of a subgroup of individuals with associated genetic features to a subgroup that did not show genetic alterations in the analyses performed. Hence there is the need to carry out specific studies on the MV autistic population, which is still poorly known, on the characteristics of this population in terms of autistic symptoms, intellectual and linguistic functioning and severity and on the association with genetic profiles.

## Data Availability

The data presented in this study are available on request from the corresponding author. The data are not publicly available due to privacy and ethical restrictions.

## References

[B1] Abdavinejad S. Charlton P. Dent M. (2024). Ultrasonic vocalizations produced by a mouse model of autism spectrum disorder differ following social experience. J. Acoust. Soc. Am. 156, A86. 10.1121/10.0035196

[B2] Abidin R. R. (1990). Manual for the parenting stress index: short form. Charlottesville, VA: Pediatric Psychology Press.

[B3] Achenbach T. M. Dumenci L. Rescorla L. A. (2001). Ratings of relations between DSM-IV diagnostic categories and items of the CBCL/6-18, TRF, and YSR. Burlington, VT: University of Vermont, 1–9.

[B4] Adviento B. Corbin I. L. Widjaja F. Desachy G. Enrique N. Rosser T. (2014). Autism traits in the RASopathies. J. Medical Genetics 51 (1), 10–20. 10.1136/jmedgenet-2013-101951 24101678 PMC4230531

[B5] Akhtar F. Bokhari S. R. A. (2025). Down syndrome. 2023 Aug 8. Treasure Island (FL): StatPearls Publishing. 30252272

[B6] Alankarage D. Szot J. O. Pachter N. Slavotinek A. Selleri L. Shieh J. T. (2020). Functional characterization of a novel PBX1 *de novo* missense variant identified in a patient with syndromic congenital heart disease. Hum. Molecular Genetics 29 (7), 1068–1082. 10.1093/hmg/ddz231 31625560 PMC7206850

[B7] Alarcón M. Abrahams B. S. Stone J. L. Duvall J. A. Perederiy J. V. Bomar J. M. (2008). Linkage, association, and gene-expression analyses identify *CNTNAP2* as an autism-susceptibility gene. Am. J. Hum. Genet. 82 (1), 150–159. 10.1016/j.ajhg.2007.09.005 18179893 PMC2253955

[B8] Alfieri P. Piccini G. Caciolo C. Perrino F. Gambardella M. L. Mallardi M. (2014). Behavioral profile in RASopathies. Am. J. Med. Genet. Part A 164 (4), 934–942. 10.1002/ajmg.a.36374 24458522

[B9] Almirall D. DiStefano C. Chang Y. C. Shire S. Kaiser A. Lu X. (2016). Longitudinal effects of adaptive interventions with a speech-generating device in minimally verbal children with ASD. J. Clinical Child Adolescent Psychology 53 (4), 442–456. 10.1080/15374416.2016.1138407 26954267 PMC4930379

[B10] American Psychiatric Association (2022). Diagnostic and statistical manual of mental disorders. 10.1176/appi.books.9780890425787

[B11] Anderson D. K. Lord C. Risi S. DiLavore P. S. Shulman C. Thurm A. (2007). Patterns of growth in verbal abilities among children with autism spectrum disorder. J. Consult. Clin. Psychol. 75 (4), 594–604. 10.1037/0022-006X.75.4.594 17663613

[B12] Angeard N. Huerta E. Jacquette A. Cohen D. Xavier J. Gargiulo M. (2018). Childhood-onset form of myotonic dystrophy type 1 and autism spectrum disorder: is there comorbidity? Neuromuscul. Disord. 28 (3), 216–221. 10.1016/j.nmd.2017.12.006 29361396

[B13] Anney R. Klei L. Pinto D. Almeida J. Bacchelli E. Baird G. (2012). Individual common variants exert weak effects on the risk for autism spectrum disorders. Hum. Molecular Genetics 21 (21), 4781–4792. 10.1093/hmg/dds301 22843504 PMC3471395

[B14] Baio J. Wiggins L. Christensen D. L. Maenner M. J. Daniels J. Warren Z. (2018). Prevalence of autism spectrum disorder among children aged 8 years - autism and developmental disabilities monitoring network, 11 sites, United States, 2014. MMWR. Surveill. Summ. 67 (6), 1–23. 10.15585/mmwr.ss6706a1 29701730 PMC5919599

[B15] Bal V. H. Katz T. Bishop S. L. Krasileva K. (2016). Understanding definitions of minimally verbal across instruments: evidence for subgroups within minimally verbal children and adolescents with autism spectrum disorder. J. Child Psychol. Psychiatry, Allied Discip. 57 (12), 1424–1433. 10.1111/jcpp.12609 27473432

[B16] Barone R. Cirnigliaro L. Saccuzzo L. Valdese S. Pettinato F. Prato A. (2023). PARK2 microdeletion in a multiplex family with autism spectrum disorder. Int. Journal Developmental Neuroscience 83 (1), 121–131. 10.1002/jdn.10246 36478299

[B17] Biesecker L. G. Johnston J. J. (2001). Greig cephalopolysyndactyly syndrome. in GeneReviews®. Editor Adam M. P. (Seattle: University of Washington). 20301619

[B18] Bird T. D. (1999). Myotonic dystrophy type 1. in GeneReviews®. Editors Adam M. P. Feldman J. Mirzaa G. M. Pagon R. A. Wallace S. E. Amemiya A. (Seattle (WA): University of Washington). 20301344

[B19] Bourgois A. Bizaoui V. Colson C. Vincent‐Devulder A. Molin A. Gérard M. (2024). Phenotypic and genotypic characterization of 1q21. 1 copy number variants: a report of 34 new individuals and literature review. Am. J. Med. Genet. Part A 194 (3), e63457. 10.1002/ajmg.a.63457 37881147

[B20] Bradbury K. Duvall S. W. Armstrong K. Hall T. A. (2022). Survey of training experiences and clinical practice in assessment for autism spectrum disorder by neuropsychologists. Clin. Neuropsychologist 36 (5), 856–873. 10.1080/13854046.2021.1948610 34308763

[B21] Braida C. Stefanatos R. K. Adam B. Mahajan N. Smeets H. J. Niel F. (2010). Variant CCG and GGC repeats within the CTG expansion dramatically modify mutational dynamics and likely contribute toward unusual symptoms in some myotonic dystrophy type 1 patients. Hum. Molecular Genetics 19 (8), 1399–1412. 10.1093/hmg/ddq015 20080938

[B22] Busch R. M. Srivastava S. Hogue O. Frazier T. W. Klaas P. Hardan A. (2019). Neurobehavioral phenotype of autism spectrum disorder associated with germline heterozygous mutations in PTEN. Transl. Psychiatry 9 (1), 253. 10.1038/s41398-019-0588-1 31594918 PMC6783427

[B23] Cao X. Li J. Song H. Zhu Y. (2021). Zhonghua Yi Xue Yi Chuan Xue Za Zhi 38 (10), 933–936. 10.3760/cma.j.cn511374-20201020-00732 34625926

[B24] Castellucci G. A. McGinley M. J. McCormick D. A. (2016). Knockout of *Foxp2* disrupts vocal development in mice. Sci. Rep. 6, 23305. 10.1038/srep23305 26980647 PMC4793191

[B25] Curatolo P. Porfirio M. C. Manzi B. Seri S. (2004). Autism in tuberous sclerosis. Eur. Journal Paediatric Neurology 8 (6), 327–332. 10.1016/j.ejpn.2004.08.005 15542389

[B26] Deriziotis P. O’Roak B. J. Graham S. A. Estruch S. B. Dimitropoulou D. Bernier R. A. (2014). *De novo* TBR1 mutations in sporadic autism disrupt protein functions. Nat. Communications 5 (1), 4954. 10.1038/ncomms5954 PMC4212638 25232744

[B27] DiStefano C. Shih W. Kaiser A. Landa R. Kasari C. (2016). Communication growth in minimally verbal children with ASD: the importance of interaction. Autism Res. 9 (10), 1093–1102. 10.1002/aur.1594 26824676

[B28] Dolcetti A. Silversides C. K. Marshall C. R. Lionel A. C. Stavropoulos D. J. Scherer S. W. (2013). 1q21. 1 microduplication expression in adults. Genet. Med. 15 (4), 282–289. 10.1038/gim.2012.129 23018752 PMC3817079

[B29] Durand C. M. Betancur C. Boeckers T. M. Bockmann J. Chaste P. Fauchereau F. (2007). Mutations in the gene encoding the synaptic scaffolding protein SHANK3 are associated with autism spectrum disorders. Nat. Genet. 39 (1), 25–27. 10.1038/ng1933 17173049 PMC2082049

[B30] Einfeld S. L. Aman M. (1995). Issues in the taxonomy of psychopathology in mental retardation. J. Autism Dev. Disord. 25 (2), 143–167. 10.1007/BF02178501 7559282

[B31] Ellis Weismer S. Kover S. T. (2015). Preschool language variation, growth, and predictors in children on the autism spectrum. J. Child Psychol. Psychiatry, Allied Discip. 56 (12), 1327–1337. 10.1111/jcpp.12406 25753577 PMC4565784

[B32] Ey E. Torquet N. de Chaumont F. Lévi-Strauss J. Ferhat A. T. Bourgeron T. (2012). Shank2 mutant mice display hyperactivity and a reduced acoustic startle response, but normal ultrasonic vocalizations. Mol. Autism 3 (1), 12. 10.1186/2040-2392-3-12 23116158 PMC3528421

[B33] Fernandez B. A. Roberts W. Chung B. Weksberg R. Meyn S. Szatmari P. (2010). Phenotypic spectrum associated with *de novo* and inherited deletions and duplications at 16p11. 2 in individuals ascertained for diagnosis of autism spectrum disorder. J. Medical Genetics 47 (3), 195–203. 10.1136/jmg.2009.069369 19755429

[B34] Fisher S. E. Scharff C. (2009). FOXP2 as a molecular window into speech and language. Trends Genet. 25 (4), 166–177. 10.1016/j.tig.2009.03.002 19304338

[B35] Fok M. Bal V. H. (2019). Differences in profiles of emotional behavioral problems across instruments in verbal *versus* minimally verbal children with autism spectrum disorder. Autism Res. 12 (9), 1367–1375. 10.1002/aur.2126 31102337 PMC6733634

[B36] Frank Y. (2021). The neurological manifestations of Phelan-McDermid syndrome. Pediatr. Neurology 122, 59–64. 10.1016/j.pediatrneurol.2021.06.002 34325981

[B37] Frazier Z. J. Kilic S. Osika H. Mo A. Quinn M. Ballal S. (2025). Novel phenotypes and genotype-phenotype correlations in a large clinical cohort of patients with kleefstra syndrome. Clin. Genetics 107, 636–645. 10.1111/cge.14697 39746677 PMC12050201

[B38] Genovese A. Butler M. G. (2023). The autism spectrum: behavioral, psychiatric and genetic associations. Genes 14 (3), 677. 10.3390/genes14030677 36980949 PMC10048473

[B39] Godfrey M. Hepburn S. Fidler D. J. Tapera T. Zhang F. Rosenberg C. R. (2019). Autism spectrum disorder (ASD) symptom profiles of children with comorbid down syndrome (DS) and ASD: a comparison with children with DS-only and ASD-Only. Res. Developmental Disabilities 89, 83–93. 10.1016/j.ridd.2019.03.003 30959431

[B40] Gomes-Pereira M. Bidichandani S. I. Monckton D. G. (2004). Analysis of unstable triplet repeats using small-pool polymerase chain reaction. Methods Molecular Biology Clift. N.J. 277, 61–76. 10.1385/1-59259-804-8:061 15201449

[B41] Goodman L. D. Cope H. Nil Z. Ravenscroft T. A. Charng W. L. Lu S. (2021). TNPO2 variants associate with human developmental delays, neurologic deficits, and dysmorphic features and alter TNPO2 activity in drosophila. Am. Journal Human Genetics 108 (9), 1669–1691. 10.1016/j.ajhg.2021.06.019 34314705 PMC8456166

[B42] Gotham K. Pickles A. Lord C. (2009). Standardizing ADOS scores for a measure of severity in autism spectrum disorders. J. Autism Dev. Disord. 39 (5), 693–705. 10.1007/s10803-008-0674-3 19082876 PMC2922918

[B43] Griffiths R. Battaglia F. M. Savoini M. Huntley M. (2007). “Griffiths mental development scales, revised: 0-2 Anni,” in Edizione italiana a cura di Francesca Maria Battaglia e Margherita Savoini. Editors Griffiths M. /R. Giunti O. S.

[B44] Gripp K. W. Weaver K. N. (2023). HRAS-related Costello syndrome. in GeneReviews®. 20301680

[B45] Guerrera S. Fucà E. Petrolo E. De Stefano A. Casula L. Logrieco M. G. (2025). Exploring the clinical features of minimally verbal autistic children. Front. Psychiatry 16, 1549092. 10.3389/fpsyt.2025.1549092 40182200 PMC11967401

[B46] Hewitt A. S. Stancliffe R. J. Sirek A. J. Hall-Lande J. Taub S. Engler J. (2012). Characteristics of adults with autism spectrum disorder who use adult developmental disability services: results from 25 US states. Res. Autism Spectr. Disord. 6 (2), 741–751. 10.1016/j.rasd.2011.10.007

[B47] Huang C.-Y. Yen H.-C. Tseng M.-H. Tung L.-C. Chen Y.-D. Chen K.-L. (2014). Impacts of autistic behaviors, emotional and behavioral problems on parenting stress in caregivers of children with autism. J. Autism Dev. Disord. 44 (6), 1383–1390. 10.1007/s10803-013-2000-y 24287878

[B48] Hus V. Gotham K. Lord C. (2014). Standardizing ADOS domain scores: separating severity of social affect and restricted and repetitive behaviors. J. Autism Dev. Disord. 44 (10), 2400–2412. 10.1007/s10803-012-1719-1 23143131 PMC3612387

[B49] Hyman S. L. Levy S. E. Myers S. M. (2020). Identification, evaluation, and management of children with autism spectrum disorder. Pediatrics 145, e20193447. 10.1542/peds.2019-3447 31843864

[B50] Kasari C. Freeman S. Paparella T. (2006). Joint attention and symbolic play in young children with autism: a randomized controlled intervention study. J. Child Psychol. Psychiatry 47 (6), 611–620. 10.1111/j.1469-7610.2005.01567.x 16712638

[B51] Kissine M. Saint-Denis A. Mottron L. (2023). Language acquisition can be truly atypical in autism: beyond joint attention. Neurosci. Biobehavioral Reviews 153, 105384. 10.1016/j.neubiorev.2023.105384 37683987

[B52] Kleefstra T. de Leeuw N. (2010). Kleefstra syndrome. in GeneReviews®. Editor Adam M. P. (Seattle: University of Washington). 20945554

[B53] Koegel L. K. Glugatch L. B. Koegel R. L. Castellon F. A. (2019). Targeting IEP social goals for children with autism in an inclusive summer camp. J. Autism Dev. Disord. 49 (6), 2426–2436. 10.1007/s10803-019-03992-4 30927180 PMC6548680

[B54] La Valle C. Shen L. Shih W. Kasari C. Shire S. Lord C. (2024). Does gestural communication influence later spoken language ability in minimally verbal autistic children? J. Speech, Language, Hearing Research 67 (7), 2283–2296. 10.1044/2024_JSLHR-23-00433 38861424 PMC11253808

[B55] Leonardi E. Cerasa A. Servidio R. Costabile A. Famà F. I. Carrozza C. (2021). The route of stress in parents of young children with and without autism: a path-analysis study. Int. J. Environ. Res. Public Health 18 (20), 10887. 10.3390/ijerph182010887 34682634 PMC8535200

[B56] Livak K. J. Schmittgen T. D. (2001). Analysis of relative gene expression data using real-time quantitative PCR and the 2(-Delta Delta C(T)) method. Methods (San Diego, Calif.) 25 (4), 402–408. 10.1006/meth.2001.1262 11846609

[B57] Loke Y. J. Hannan A. J. Craig J. M. (2015). The role of epigenetic change in autism spectrum disorders. Front. Neurology 6, 107. 10.3389/fneur.2015.00107 26074864 PMC4443738

[B58] Lord C. Risi S. Lambrecht L. Cook E. H. Leventhal B. L. DiLavore P. C. (2000). The autism diagnostic observation schedule—generic: a standard measure of social and communication deficits associated with the spectrum of autism. J. Autism Dev. Disord. 30, 205–223. 10.1023/A:1005592401947 11055457

[B59] Lord C. Rutter M. DiLavore P. Risi S. Gotham K. Bishop S. (2012). Autism diagnostic observation schedule–2nd edition (ADOS-2). Los Angeles, CA: Western Psychological Corporation.

[B60] Lowenthal R. Paula C. S. Schwartzman J. S. Brunoni D. Mercadante M. T. (2007). Prevalence of pervasive developmental disorder in Down’s syndrome. J. Autism Developmental Disorders 37, 1394–1395. 10.1007/s10803-007-0374-4 17410415

[B61] Luyster R. J. Kadlec M. B. Carter A. Tager-Flusberg H. (2008). Language assessment and development in toddlers with autism spectrum disorders. J. Autism Dev. Disord. 38 (8), 1426–1438. 10.1007/s10803-007-0510-1 18188685

[B62] Maenner M. J. Warren Z. Williams A. R. Amoakohene E. Bakian A. V. Bilder D. A. (2023). Prevalence and characteristics of autism spectrum disorder among children aged 8 years - autism and developmental disabilities monitoring network, 11 sites, United States, 2020. MMWR. Surveill. Summ. 72 (2), 1–14. 10.15585/mmwr.ss7202a1 36952288 PMC10042614

[B63] Mandy W. Lai M. C. (2016). Annual research review: the role of the environment in the developmental psychopathology of autism spectrum condition. J. Child Psychology Psychiatry, Allied Disciplines 57 (3), 271–292. 10.1111/jcpp.12501 26782158

[B65] Moss J. Richards C. Nelson L. Oliver C. (2013). Prevalence of autism spectrum disorder symptomatology and related behavioural characteristics in individuals with Down syndrome. Autism 17 (4), 390–404. 10.1177/1362361312442790 22589453

[B66] Mottron L. Gagnon D. (2023). Prototypical autism: new diagnostic criteria and asymmetrical bifurcation model. Acta Psychologica 237, 103938. 10.1016/j.actpsy.2023.103938 37187094

[B67] Ng R. Kalinousky A. J. Harris J. (2024). Neuropsychological profile associated with KAT6A syndrome: emergent genotype-phenotype trends. Orphanet Journal Rare Diseases 19 (1), 196. 10.1186/s13023-024-03175-0 38741077 PMC11092058

[B68] Norrelgen F. Fernell E. Eriksson M. Hedvall Å. Persson C. Sjölin M. (2015). Children with autism spectrum disorders who do not develop phrase speech in the preschool years. Autism 19 (8), 934–943. 10.1177/1362361314556782 25488002

[B69] Northrup H. Koenig M. K. Pearson D. A. Au K. S. (1999). Tuberous sclerosis complex. in GeneReviews®. Editor Adam M. P. (Seattle: University of Washington). 20301399

[B70] Oakland T. Kreutzer J. S. DeLuca J. Caplan B. (2011). “Adaptive behavior assessment system,” in Encyclopedia of clinical neuropsychology (New York, NY: Springer).

[B71] Oberman L. M. Boccuto L. Cascio L. Sarasua S. Kaufmann W. E. (2015). Autism spectrum disorder in Phelan-McDermid syndrome: initial characterization and genotype-phenotype correlations. Orphanet Journal Rare Diseases 10, 1–9. 10.1186/s13023-015-0323-9 26306707 PMC4549933

[B72] Oxelgren U. W. Aberg M. Myrelid A. Anneren G. Westerlund J. Gustafsson J. (2019). Autism needs to be consid-ered in children with Down syndrome. Acta Paediatr. 108 (11), 2019–2026. 10.1111/apa.14850 31090964

[B73] Papanikolaou K. Ntre V. Gertsou I.-M. Tagkouli E. Tzavara C. Pehlivanidis A. (2022). Parenting children with autism spectrum disorder during crises: differential responses between the financial and the COVID-19 pandemic crisis. J. Clin. Med. 11 (5), 1264. 10.3390/jcm11051264 35268354 PMC8911193

[B74] Pascual-Morena C. Martínez-Vizcaíno V. Cavero-Redondo I. Álvarez-Bueno C. Lucerón-Lucas-Torres M. Saz-Lara A. (2024). A meta-analysis of the prevalence of neuropsychiatric disorders and their association with disease onset in myotonic dystrophy. Acta Neuropsychiatr., 1–12. 10.1017/neu.2024.27 39376198

[B75] Pecukonis M. Plesa Skwerer D. Eggleston B. Meyer S. Tager-Flusberg H. (2019). Concurrent social communication predictors of expressive language in minimally verbal children and adolescents with autism spectrum disorder. J. Autism Dev. Disord. 49 (9), 3767–3785. 10.1007/s10803-019-04089-8 31187332 PMC6988896

[B76] Peñagarikano O. Abrahams B. S. Herman E. I. Winden K. D. Gdalyahu A. Dong H. (2011). Absence of *Cntnap2* leads to epilepsy, neuronal migration abnormalities, and core autism-related deficits. Cell 147 (1), 235–246. 10.1016/j.cell.2011.08.040 21962519 PMC3390029

[B77] Phelan K. Rogers R. C. Boccuto L. (2005). “Phelan-McDermid Syndrome-*SHANK3* related,” in GeneReviews®. Editor Adam M. P. (Seattle: University of Washington). 20301377

[B78] Pickles A. Anderson D. K. Lord C. (2014). Heterogeneity and plasticity in the development of language: a 17-year follow-up of children referred early for possible autism. J. Child Psychol. Psychiatry 55 (12), 1446–1456. 10.1111/jcpp.12269 24889883

[B79] Prasad C. Prasad A. N. Chodirker B. N. Lee C. Dawson A. K. Jocelyn L. J. (2000). Genetic evaluation of pervasive developmental disorders: the terminal 22q13 deletion syndrome May represent a recognizable phenotype. Clin. Genetics 57 (2), 103–109. 10.1034/j.1399-0004.2000.570203.x 10735630

[B80] Quesnel-Vallières M. Weatheritt R. J. Cordes S. P. Blencowe B. J. (2019). Autism spectrum disorder: insights into convergent mechanisms from transcriptomics. Nat. Reviews. Genet. 20 (1), 51–63. 10.1038/s41576-018-0066-2 30390048

[B81] Rankine J. Li E. Lurie S. Rieger H. Fourie E. Siper P. M. (2017). Language ENvironment analysis (LENA) in Phelan-McDermid syndrome: validity and suggestions for use in minimally verbal children with autism spectrum disorder. J. Autism Developmental Disorders 47 (6), 1605–1617. 10.1007/s10803-017-3082-8 28255759 PMC6196360

[B82] Richards S. Aziz N. Bale S. Bick D. Das S. Gastier-Foster J. (2015). Standards and guidelines for the interpretation of sequence variants: a joint consensus recommendation of the American college of medical genetics and genomics and the association for molecular pathology. Genet. Medicine 17 (5), 405–424. 10.1038/gim.2015.30 25741868 PMC4544753

[B83] Riggs E. R. Andersen E. F. Cherry A. M. Kantarci S. Kearney H. Patel A. (2021). Technical standards for the interpretation and reporting of constitutional copy-number variants: a joint consensus recommendation of the American college of medical genetics and genomics (ACMG) and the clinical genome resource (ClinGen). Genet. Med. 23 (11), 2230–2257. 10.1038/s41436-019-0686-8 33731880

[B84] Roid G. H. Koch C. (2017). “Leiter-3: nonverbal cognitive and neuropsychological assessment,” in Handbook of nonverbal assessment, 127–150.

[B85] Roid G. H. Miller L. Press J. (2000). Leiter international performance scale-revised (Leiter-R). Illinois: Stoelting Co.

[B86] Rutter M. (2011). A selective scientific history of autism. in Textbook of autism spectrum disorders. Editors Hollander E. Kolevzon A. Coyle J. T. (American Psychiatric Publishing, Inc), 5–21.

[B87] Rutter M. Le Couteur A. Lord C. (2003). ADI-R autism diagnostic interview-revised (ADI-R). Los Angeles, CA: Western Psychological Services.

[B88] Rutter M. Moffitt T. E. Caspi A. (2006). Gene-environment interplay and psychopathology: multiple varieties but real effects. J. Child Psychology Psychiatry, Allied Disciplines 47 (3-4), 226–261. 10.1111/j.1469-7610.2005.01557.x 16492258

[B89] Samanta D. (2020). An updated review of tuberous sclerosis complex-associated autism spectrum disorder. Pediatr. Neurology 109, 4–11. 10.1016/j.pediatrneurol.2020.03.008 32563542

[B90] Sanders S. J. Ercan-Sencicek A. G. Hus V. Luo R. Murtha M. T. Moreno-De-Luca D. (2011). Multiple recurrent *de novo* CNVs, including duplications of the 7q11.23 Williams syndrome region, are strongly associated with autism. Neuron 70 (5), 863–885. 10.1016/j.neuron.2011.05.002 21658581 PMC3939065

[B91] Sandin S. Lichtenstein P. Kuja-Halkola R. Larsson H. Hultman C. M. Reichenberg A. (2014). The familial risk of autism. JAMA 311 (17), 1770–1777. 10.1001/jama.2014.4144 24794370 PMC4381277

[B92] Santoro J. D. Pagarkar D. Chu D. T. Rosso M. Paulsen K. C. Levitt P. (2021). Neurologic complications of Down syndrome: a systematic review. J. Neurology 268 (12), 4495–4509. 10.1007/s00415-020-10179-w 32920658

[B93] Savatt J. M. Myers S. M. (2021). Genetic testing in neurodevelopmental disorders. Front. Pediatrics 9, 526779. 10.3389/fped.2021.526779 33681094 PMC7933797

[B94] Schaeffer J. Abd El-Raziq M. Castroviejo E. Durrleman S. Ferré S. Grama I. (2023). Language in autism: domains, profiles and co-occurring conditions. J. Neural Transm. (Vienna) 130, 433–457. 10.1007/s00702-023-02592-y 36922431 PMC10033486

[B95] Scibelli F. Fucà E. Guerrera S. Lupi E. Alfieri P. Valeri G. (2021). Clinical and individual features associated with maternal stress in young adolescents with autism spectrum disorder. Autism Res. 14 (9), 1935–1947. 10.1002/aur.2539 34013607

[B96] Siracusano M. Riccioni A. Baratta A. Baldi M. Curatolo P. Mazzone L. (2019). Autistic symptoms in Greig cephalopolysyndactyly syndrome: a family case report. J. Medical Case Reports 13 (1), 100. 10.1186/s13256-019-2043-6 31010437 PMC6477736

[B97] Slavotinek A. Risolino M. Losa M. Cho M. T. Monaghan K. G. Schneidman-Duhovny D. (2017). *De novo,* deleterious sequence variants that alter the transcriptional activity of the homeoprotein PBX1 are associated with intellectual disability and pleiotropic developmental defects. Hum. Molecular Genetics 26 (24), 4849–4860. 10.1093/hmg/ddx363 29036646 PMC6455034

[B98] Slušná D. Rodríguez A. Salvadó B. Vicente A. Hinzen W. (2021). Relations between language, non-verbal cognition, and conceptualization in non- or minimally verbal individuals with ASD across the lifespan. Autism & Dev. Lang. Impair. 6, 23969415211053264. 10.1177/23969415211053264 36440372 PMC9685121

[B99] Smalley S. L. (1998). Autism and tuberous sclerosis. J. Autism Developmental Disorders 28, 407–414. 10.1023/a:1026052421693 9813776

[B100] Styles M. Alsharshani D. Samara M. Alsharshani M. Khattab A. Qoronfleh M. W. (2020). Risk factors, diagnosis, prognosis and treatment of autism. Front. Biosci. Landmark Ed. 25 (9), 1682–1717. 10.2741/4873 32472753

[B101] Sztainberg Y. Zoghbi H. Y. (2016). Lessons learned from studying syndromic autism spectrum disorders. Nat. Neuroscience 19 (11), 1408–1417. 10.1038/nn.4420 27786181

[B102] Tager-Flusberg H. Kasari C. (2013). Minimally verbal school-aged children with autism spectrum disorder: the neglected end of the spectrum. Autism Res. 6 (6), 468–478. 10.1002/aur.1329 24124067 PMC3869868

[B103] Tassano E. Severino M. Rosina S. Papa R. Tortora D. Gimelli G. (2016). Interstitial *de novo* 18q22. 3q23 deletion: clinical, neuroradiological and molecular characterization of a new case and review of the literature. Mol. Cytogenet. 9, 1–7. 10.1186/s13039-016-0285-1 27766118 PMC5057431

[B104] Taylor C. M. Smith R. Lehman C. Mitchel M. W. Singer K. Weaver W. C. (2021). 16p11. 2 recurrent deletion. 20301775

[B105] Tek S. Mesite L. Fein D. Naigles L. (2014). Longitudinal analyses of expressive language development reveal two distinct language profiles among young children with autism spectrum disorders. J. Autism Developmental Disorders 44 (1), 75–89. 10.1007/s10803-013-1853-4 23719855 PMC4557801

[B106] Thapar A. Rutter M. (2021). Genetic advances in autism. J. Autism Developmental Disorders 51 (12), 4321–4332. 10.1007/s10803-020-04685-z 32940822 PMC8531042

[B107] Thurm A. Lord C. Lee L. C. Newschaffer C. (2007). Predictors of language acquisition in preschool children with autism spectrum disorders. J. Autism Dev. Disord. 37 (9), 1721–1734. 10.1007/s10803-006-0300-1 17180717

[B108] Thurm A. Manwaring S. S. Swineford L. Farmer C. (2015). Longitudinal study of symptom severity and language in minimally verbal children with autism. J. Child Psychol. Psychiatry, Allied Discip. 56 (1), 97–104. 10.1111/jcpp.12285 24961159 PMC4581593

[B109] Thurm A. Farmer C. Salzman E. Lord C. Bishop S. (2019). State of the field: differentiating intellectual disability from autism spectrum disorder. Front. Psychiatry 10, 526. 10.3389/fpsyt.2019.00526 31417436 PMC6683759

[B110] Tischkowitz M. Colas C. Pouwels S. Hoogerbrugge N. PHTS Guideline Development Group European Reference Network GENTURIS (2020). Cancer surveillance guideline for individuals with PTEN hamartoma tumour syndrome. Eur. Journal Human Genetics 28 (10), 1387–1393. 10.1038/s41431-020-0651-7 32533092 PMC7608293

[B111] van der Plas E. Hamilton M. J. Miller J. N. Koscik T. R. Long J. D. Cumming S. (2019). Brain structural features of myotonic dystrophy type 1 and their relationship with CTG repeats. J. Neuromuscular Diseases 6 (3), 321–332. 10.3233/JND-190397 31306140 PMC7480174

[B112] Vargha-Khadem F. Gadian D. G. Copp A. Mishkin M. (2005). FOXP2 and the neuroanatomy of speech and language. Nat. Rev. Neurosci. 6 (2), 131–138. 10.1038/nrn1605 15685218

[B113] Vermeulen K. de Boer A. Janzing J. G. Koolen D. A. Ockeloen C. W. Willemsen M. H. (2017). Adaptive and maladaptive functioning in kleefstra syndrome compared to other rare genetic disorders with intellectual disabilities. Am. Journal Medical Genetics Part A 173 (7), 1821–1830. 10.1002/ajmg.a.38280 28498556

[B114] Vicari S. Napoli E. Cordeddu V. Menghini D. Alesi V. Loddo S. (2019). Copy number variants in autism spectrum disorders. Prog. Neuro-Psychopharmacology & Biological Psychiatry 92, 421–427. 10.1016/j.pnpbp.2019.02.012 30797015

[B115] Vogindroukas I. Stankova M. Chelas E. N. Proedrou A. (2022). Language and speech characteristics in autism. Neuropsychiatr. Dis. Treat. 18, 2367–2377. 10.2147/NDT.S331987 36268264 PMC9578461

[B116] Vorstman J. A. S. Scherer S. W. (2023). Contemplating syndromic autism. Genetics in medicine. Official Journal Am. Coll. Med. Genet. 25 (10), 100919. 10.1016/j.gim.2023.10091 37330697

[B117] Vorstman J. A. S. Parr J. R. Moreno-De-Luca D. Anney R. J. L. Nurnberger J. I. Jr. Hallmayer J. F. (2017). Autism genetics: opportunities and challenges for clinical translation. Nat. Reviews. Genet. 18 (6), 362–376. 10.1038/nrg.2017.4 28260791

[B118] Weiner D. J. Wigdor E. M. Ripke S. Walters R. K. Kosmicki J. A. Grove J. (2017). Polygenic transmission disequilibrium confirms that common and rare variation act additively to create risk for autism spectrum disorders. Nat. Genet. 49 (7), 978–985. 10.1038/ng.3863 28504703 PMC5552240

[B119] Weiss L. A. Shen Y. Korn J. M. Arking D. E. Miller D. T. Fossdal R. (2008). Association between microdeletion and microduplication at 16p11.2 and autism. N. Engl. Journal Medicine 358 (7), 667–675. 10.1056/NEJMoa075974 18184952

[B120] Weiss L. A. Arking D. E. Daly M. J. Chakravarti A. (2009). A genome-wide linkage and association scan reveals novel loci for autism. Nature 461 (7265), 802–808. 10.1038/nature08490 19812673 PMC2772655

[B121] Williams D. L. Siegel M. Mazefsky C. A. Autism and Developmental Disorders Inpatient Research Collaborative ADDIRC (2018). Problem behaviors in autism spectrum disorder: association with verbal ability and adapting/coping skills. J. Autism Dev. Disord. 48 (11), 3668–3677. 10.1007/s10803-017-3179-0 28597186 PMC5924584

[B122] Wodka E. L. Mathy P. Kalb L. (2013). Predictors of phrase and fluent speech in children with autism and severe language delay. Pediatrics 131 (4), e1128–e1134. 10.1542/peds.2012-2221 23460690 PMC9923624

[B123] Xavier J. Zhou B. Bilan F. Zhang X. Gilbert-Dussardier B. Viaux-Savelon S. (2018). 1q21.1 microduplication: large verbal-nonverbal performance discrepancy and ddPCR assays of HYDIN/HYDIN2 copy number. NPJ Genomic Medicine 3, 24. 10.1038/s41525-018-0059-2 30155272 PMC6105585

[B124] Yehia L. Keel E. Eng C. (2020). The clinical spectrum of *PTEN* mutations. Annu. Review Medicine 71, 103–116. 10.1146/annurev-med-052218-125823 31433956

[B125] Zafeiriou D. I. Ververi A. Dafoulis V. Kalyva E. Vargiami E. (2013). Autism spectrum disorders: the quest for genetic syndromes. Am. Journal Medical Genetics 162B (4), 327–366. 10.1002/ajmg.b.32152 23650212

[B126] Zeidan J. Fombonne E. Scorah J. Ibrahim A. Durkin M. S. Saxena S. (2022). Global prevalence of autism: a systematic review update. Autism Res. 15 (5), 778–790. 10.1002/aur.2696 35238171 PMC9310578

[B127] Ziats C. A. Patterson W. G. Friez M. (2021). Syndromic autism revisited: review of the literature and lessons learned. Pediatr. Neurol. 114, 21–25. 10.1016/j.pediatrneurol.2020.06.011 33189026

